# Reconstruction Using Conventional Implants in a Patient With an Acetabular Fracture Due to Cervical Cancer Metastasis: A Case Report

**DOI:** 10.7759/cureus.112600

**Published:** 2026-07-13

**Authors:** Vicente Portilla, Francisco Mahaluf Recasens, Luis Bahamonde

**Affiliations:** 1 Medicine Department, San Sebastián University, Concepción, CHL; 2 Hip Unit, Complejo Asistencial Dr. Víctor Ríos Ruiz, Los Ángeles, CHL; 3 Orthopaedic Department, Universidad de Chile, Santiago, CHL

**Keywords:** acetabular metastasis, acetabular reconstruction, bone metastasis, cemented total hip arthroplasty, orthopedic oncology, palliative surgery, surgical case report, uterine cervical cancer

## Abstract

Metastases in the acetabular area pose significant difficulties for management. Lytic lesions associated with pathological fractures lead to pain and disability. In patients with limited survival, reconstructive surgery may allow swift functional recovery and pain relief.

Reconstructive techniques vary from percutaneous insertion of screws to complex reconstructions by means of personalized 3D-printed implants. On the other hand, osseous metastases from cervical cancer are infrequent and lytic in nature, and reports on surgical management are scarce.

We present the case of a 37-year-old female patient with a pathological fracture and severe osteolysis in the acetabular area, due to metastases from cervical cancer. Reconstruction was undertaken with the use of regular implants common to endoprosthetic replacements, along with the reinforcement of the osseous bed with hydroxyapatite-coated Schanz pins and an acetabular cage. Post-operative improvement in the modified Harris Hip Score in this patient supports this approach as a cost-effective method.

## Introduction

Osseous metastases from cervical cancer are uncommon, ranging from 5.2% to 6.6% [1,2], although the consequences are similar to those from other tumors associated with lytic lesions [3]. Studies focusing on skeletal metastases from this origin are scarce, requiring multi-institutional efforts to provide more valid conclusions [2]. Moreover, surgical treatment is reported in only a few cases, in some without a specific description of procedures [1,2]. Aside from this, since many cases of cervical cancer include radiotherapy as part of the treatment protocols, secondary skeletal consequences (radiation-induced osteonecrosis) could be contributing to the occurrence of pathological fractures in pelvic bones [4].

Overall, surgical management of acetabular metastases varies from percutaneous insertion of screws along pelvic corridors for mechanical reinforcement, cementation of contained defects, resections and reconstruction utilizing implants regularly suited for revision surgery, and personalized 3D-printed endoprostheses [5-7]. The method popularized by Harrington [8] consists of mechanical reinforcement of the remaining bone by means of insertion of threaded pins that cross the ilium to the acetabular dome and floor, allowing the placement of a cemented endoprosthesis, and has proven effective for the management of pain and restoration of function in selected patients. Modifications of such methods have been reported, attempting to improve the strength of the construct [9,10].

We present the case of a patient with metastases from cervical cancer and extensive involvement of the supraacetabular area and acetabular floor. There were a pathological fracture and moderate protrusion. For the reconstruction, we performed a modification of the Harrington technique, with cementation of an acetabular cage and endoprosthetic reconstruction of the hip, resulting in excellent pain relief and swift functional recovery.

## Case presentation

A 37-year-old female patient was admitted, wheelchair-bound, due to a history of progressive pain in the right hip for three months. Pain intensity increased to 10 out of 10 according to the visual analog scale (VAS), leading to an inability to stand and walk. She was previously treated for cervical cancer, including chemotherapy and radiotherapy.

**Figure 1:**
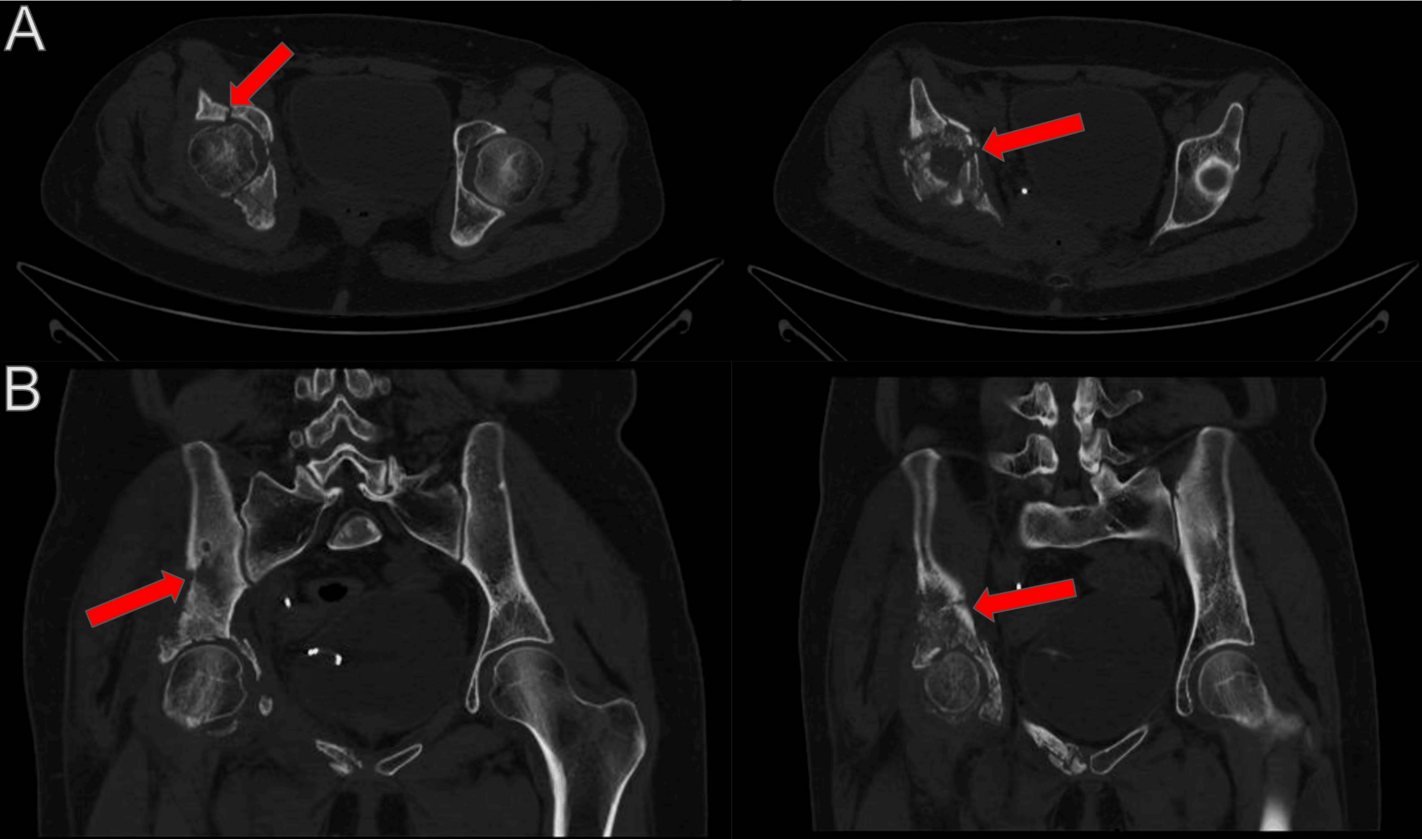
Contrast-enhanced CT of the pelvis Contrast-enhanced pelvic CT: axial views (A) and coronal views (B) showing extensive bone destruction involving the acetabular floor, supraacetabular area, and anterior wall and column (red arrows).

CT imaging showed a fracture and moderate protrusion of the right acetabulum, extensively involved with metastatic lytic lesions, including the supraacetabular area, anterior wall and column, as well as part of the iliac wing (Enneking zones I, II, and III) (Figure 1).

After a thorough assessment of the case, and despite a poor prognosis, surgical management and reconstruction of the hip were planned. The iliac wing was the only structure without obvious tumor involvement and was considered sufficient for attempting an endoprosthetic reconstruction based on the technique popularized by Harrington [8].

The patient was positioned in a "sloppy lateral" position. An extended iliofemoral approach was performed, with ample exposure of the iliac bone, after partial detachment of the gluteus medius laterally and accessing the retroperitoneal space medially. The sartorius and partially the rectus femoris were also detached from the ilium. The hip capsule was exposed superiorly, anteriorly, and posteriorly. Capsulectomy was performed, followed by the anterior dislocation of the femoral head and osteotomy at the base of the neck, as done regularly for a primary stem insertion.

**Figure 2:**
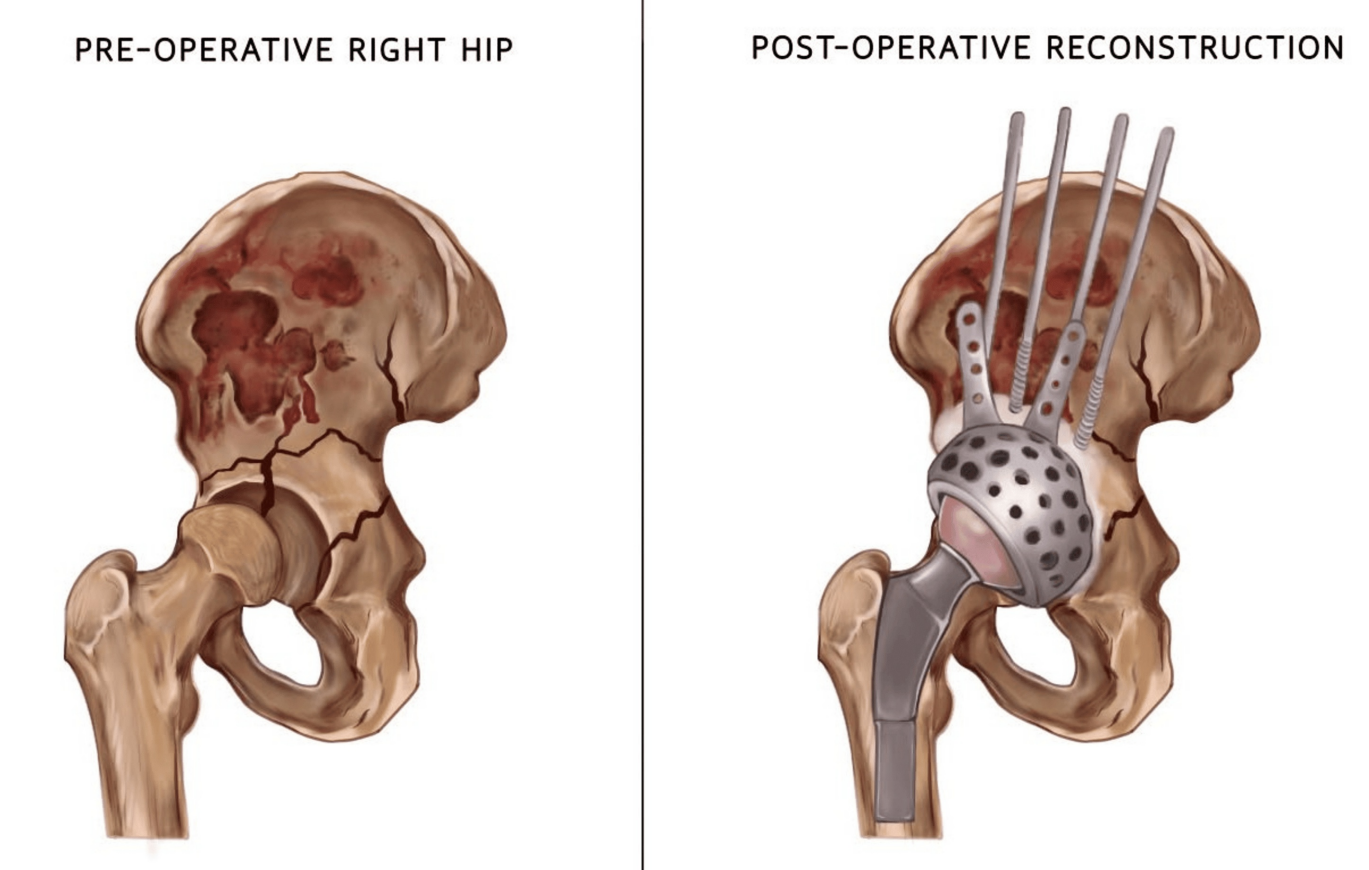
Schematic representation of the modified Harrington reconstruction for the right acetabulum (Left) Pre-operative right hip demonstrating a large secondary osteolytic defect in the supraacetabular region of the ilium, associated with a pathological fracture. (Right) Post-operative skeletal reconstruction utilizing four Schanz pins anchored in the intact ilium to reinforce the construct. A Restoration GAP II Acetabular Revision Cage (Stryker, Kalamazoo, MI, USA) is cemented, with its superior fixation tabs secured into the bone. The joint is restored with a polyethylene cup and a prosthetic femoral stem. This illustration was created using ibis Paint X (ibis Inc., Tokyo, Japan) by illustrator Agustina Rivera Espinoza.

Through complete visualization of the acetabulum, curettage of the tumor was performed, leaving a shell of bone from the acetabular rim and a wide defect in the supraacetabular area. Four hydroxyapatite-coated Schanz pins were inserted in an antegrade fashion from the iliac wing, pointing to the acetabular floor and posterior and anterior walls. A cutter was used to trim the pins at the level of the iliac crest. A properly sized acetabular cage was tested (Restoration Gap II, Stryker, Kalamazoo, MI, USA, 52 mm in diameter), with careful assessment of orientation for further cup insertion. Contact of the tip of the Schanz pins with the cage was done on purpose, as an attempt to increase the support of the construct. Cementation of the supraacetabular bony cavity and the remaining acetabulum was performed, followed by the insertion of the acetabular cage. Additional fixation was achieved by screws from the ring holes towards the cemented supraacetabular area. Cementation of a polyethylene cup was completed, carefully assessing proper orientation for hip stability. Through the same approach, intermuscular dissection lateral to the rectus femoris allowed good exposure of the femoral neck. A cemented Exeter stem was implanted, and the hip was then reduced, confirming good stability and the absence of impingement of the neck of the stem with the rim of the cup and/or cage (Figure 2). Two grams of vancomycin powder were used prior to wound closure, which was done by suturing the fascia of the abdominal wall to the fascia of the gluteus medius with Vicryl 1, extending distally to include the fascia from the rectus femoris.

**Figure 3:**
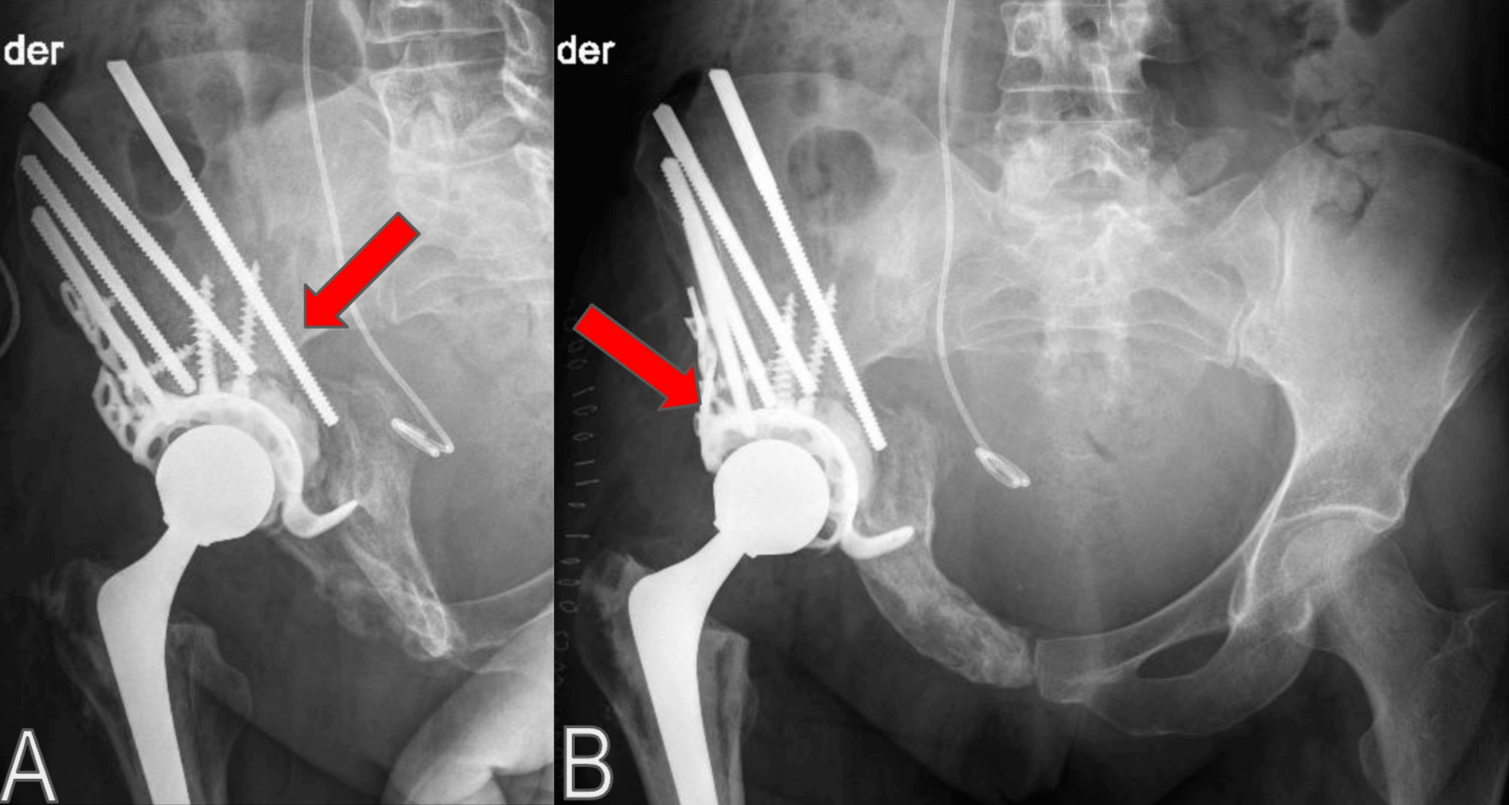
Post-operative radiographs Alar (A) and anteroposterior (B) views showing the final reconstruction, with the distribution of the four hydroxyapatite-coated Schanz pins and acetabular cage, and reinforcement of the weight-bearing area with screws and cement (red arrows).

Post-operatively, the patient immediately began physical therapy, being able to ambulate with crutches on the first post-operative day. At six months of follow-up, the patient was free of pain, ambulating with one crutch, and her modified Harris Hip Score improved to 91 points (Figure 3).

## Discussion

Metastatic involvement of the acetabular region is associated with pain and disability, which severely deteriorates the often-limited lifespan of these already-fragile patients. Primary acetabular tumors require, when feasible, complex reconstructions, associated with numerous complications including infection, dislocation, and tumor progression [5,11,12]. Reconstructive methods vary from none to 3D-printed personalized implants.

Metastatic lesions, especially those associated with pathological fractures of the acetabulum, with or without the involvement of other zones according to the Enneking classification, require a careful and multidisciplinary evaluation. Factors such as overall prognosis, type of primary tumor, extent of the lesion, patient performance status, general medical condition, and the expertise of the surgical team must be considered to decide whether surgical management could be of benefit, as well as in the selection of a particular technique. Few reports and studies on pelvic bone metastases from cervical cancer have been published. In a multicenter study from Japan, only two of 75 patients underwent surgical treatment, but there is no description of the procedure [2]. Additionally, most patients received radiotherapy. When considering the etiology of the bone-destructive process, together with metastatic invasion, the effect of radiotherapy must also be kept in mind [4]. In the patient presented here, despite a poor prognosis, her age, symptom severity, and disability favored surgical treatment. The limited viable bone remaining for reconstruction was a major concern and a critical factor in selecting the method.

The technique advocated by Harrington has proven effective as a reconstruction method for these conditions, providing a stable construct to allow prompt functional recovery [8]. Technical modifications have been reported, attempting to improve the mechanical capabilities for early weight-bearing [9,10]. In our patient, we used hydroxyapatite-coated Schanz pins as a method to improve fixation to the remaining iliac bone, a technique previously reported by Escudero-Acurio et al. and Ríos Moreno et al. [13,14]. Contact between the tips of the pins and the acetabular cage, integrating the construct together with cement, may help to provide additional strength. Overall, the treatment strategy and surgical technique utilized for this patient were rewarding. A swift recovery was accomplished, with excellent pain relief and a return to walking capacity. Therefore, even in cases with extensive bone damage secondary to metastases, this method is a valuable alternative for hip joint reconstruction.

## Conclusions

The management of advanced periacetabular metastatic disease, particularly in young patients, requires a multidisciplinary approach aimed at the early restoration of quality of life and pain relief. This case presents a patient with an acetabular fracture secondary to cervical cancer metastasis who was treated using a functional salvage reconstructive technique. The use of conventional orthopedic implants (hydroxyapatite-coated Schanz pins, bone cement, and an acetabular cage) proved to be a viable method for achieving immediate biomechanical stabilization and restoring the integrity of the hip joint.

This report highlights that, through careful anatomical planning and solid biomechanical principles, it is possible to provide an effective, reproducible, and cost-effective alternative. However, the successful execution of this procedure demands a high level of technical expertise from the surgical team.

## References

[1] Matsuyama T, Tsukamoto N, Imachi M, Nakano H (1989). Bone metastasis from cervix cancer. Gynecol Oncol.

[2] Kanayama T, Mabuchi S, Shimura K, Hisamatsu T, Isohashi F, Hamasaki T, Kimura T (2015). Prognostic factors for survival in cervical cancer patients with bone metastasis. Eur J Gynaecol Oncol.

[3] Lavignac P, Prieur J, Fabre T (2020). Surgical treatment of peri-acetabular metastatic disease: retrospective, multicentre study of 91 THA cases. Orthop Traumatol Surg Res.

[4] Zhong X, Dong T, Tan Y (2020). Pelvic insufficiency fracture or bone metastasis after radiotherapy for cervical cancer? The added value of DWI for characterization. Eur Radiol.

[5] Spinelli MS, Ziranu A, Piccioli A, Maccauro G (2016). Surgical treatment of acetabular metastasis. Eur Rev Med Pharmacol Sci.

[6] Huang X, Huang D, Lin N, Yan X, Qu H, Ye Z (2025). 3D-printed prosthesis with an articular interface for anatomical acetabular reconstruction after type I + II (+ III) internal hemipelvectomy: clinical outcomes and finite element analysis. J Bone Joint Surg Am.

[7] Di Laura A, Henckel J, Hart A (2023). Custom 3D-printed implants for acetabular reconstruction: intermediate-term functional and radiographic results. JB JS Open Access.

[8] Harrington KD (1981). The management of acetabular insufficiency secondary to metastatic malignant disease. J Bone Joint Surg Am.

[9] Chimutengwende-Gordon M, Coomber R, Peat F, Tarazi N, Chou D, Carrothers A (2022). The Harrington plus reconstruction for pelvic and acetabular metastases. J Bone Oncol.

[10] Gusho CA, Chapman R, Blank AT (2020). A modified Harrington technique for periacetabular reconstruction in advanced metastatic bone disease and a discussion of alternative treatment options. Orthop Rev (Pavia).

[11] Wood TJ, Racano A, Yeung H, Farrokhyar F, Ghert M, Deheshi BM (2014). Surgical management of bone metastases: quality of evidence and systematic review. Ann Surg Oncol.

[12] Issack PS, Kotwal SY, Lane JM (2013). Management of metastatic bone disease of the acetabulum. J Am Acad Orthop Surg.

[13] Escudero-Acurio P, Mahaluf F, Bahamonde L (2022). Reconstruction of type I-II internal hemipelvectomy in a patient with pelvic myxoid chondrosarcoma: a case report. Cureus.

[14] Ríos Moreno DI, Mahaluf Recasens F, Bahamonde L (2025). Acetabular reconstruction in a case of chondrosarcoma. Cureus.

